# Caliper navigation for craniotomy planning of convexity targets

**DOI:** 10.1371/journal.pone.0251023

**Published:** 2021-05-20

**Authors:** Max Jägersberg, Michael Kosterhon, Florian Ringel

**Affiliations:** Department of Neurosurgery, University Medical Center Mainz, Johannes Gutenberg University, Mainz, Germany; I.R.C.C.S. San Raffaele Scientific Institute, Vita-Salute San Raffaele University, ITALY

## Abstract

**Introduction:**

A technique to localize a radiological target on the head convexity fast and with acceptable precision is sufficient for surgeries of superficial intracranial lesions, and of help in the setting of emergency surgery, computer navigation breakdown, limited resources and education.

We present a caliper technique based on fundamental geometry, with inexpensive and globally available tools (conventional CT or MRI image viewer, calculator, caliper).

**Methods:**

The distances of the radiological target from two landmarks (nasion and porus acusticus externus) are assessed with an image viewer and Pythagoras’ theorem. The two distances are then marked around the landmarks onto the head of the patient with help of a caliper. The intersection defines the target.

We tested the technique in a saw bone skull model and afterwards in the operating room. Convexity targets were localized with the caliper navigation technique and then with computer navigation as ground truth.

**Results:**

In the saw bone model, the mean offset between the caliper navigated target and the real target was 2.9 ± 2.8 mm, 95% CI (1.6 mm; 4.2 mm). The mean offset between computer navigated target and real target was 1.6 ± 0.9 mm, 95% CI (1.2 mm; 2 mm) (ns).

In 15 patients undergoing navigated cranial procedures, 100 targets were assessed in reference to computer navigation. The mean offset of the caliper navigation was 11 ± 5.2 mm, 95% CI (9.9 mm; 12 mm).

**Conclusion:**

This is a low-tech approach for translation of a radiological target to the patient’s head in short time and with globally available inexpensive tools, with satisfying precision for many procedures.

## Introduction

Transfer of target location from the radiological dataset to the patients’ head is a key step for performing adequately localized craniotomies for neurosurgical procedures. As long as lesions come close to or are in contact with identifiable anatomical landmarks as the bony skull base, falx, or tentorium, these structures can be used to perform standard craniotomies and follow anatomical routes for orientation. In contrast, although technically very easy to perform, craniotomies for lesions of the convexity can be challenging concerning their correct localization. The spherical neurocranium renders orientation difficult and requires a precise spatial sense to place adequate freehand convexity craniotomies. Image guidance or computer navigation have come to great help for this problem, and owing to these high-end tools, precise craniotomy planning is no longer an issue in today neurosurgery. However, the use of navigation has become routine to such an extent that if left without it, many surgeons are lacking experience and means to precisely locate a surgical convexity lesion on the head of the patient.

Computer navigation has become a standard tool with low per case costs-yet immense costs of acquisition-in present neurosurgery. However, the setup is somewhat time-consuming, requires specific imaging datasets and therefore, the use in emergency situations remains limited. Furthermore, computer navigation might not always be available in every situation at every place for the planning of craniotomies and alternative strategies are needed to place an adequate craniotomy over a convexity lesion.

To the knowledge of the authors, different approximate localization approaches based on distance measurements from landmarks are practiced by our peers, but most of them have shortcomings with respect to spherical geometry and hence precision. Lack of precision leads either to a misplaced craniotomy, rendering surgery in the best case less elegant, in the worst case leading to serious complications, or—by compensating the lack of precision—to craniotomies larger than necessary, equally not acceptable.

Here, we present a caliper-based navigation technique and its evaluation, that allows for precise localization of a chosen target from a basic non-3D CT or MRI scan onto the patient’s head, without need for further image acquisition or data processing.

## Material and methods

The clinical validation concept was approved by the local ethics committee board responsible for our Institution (Ethik-Kommission Ärztekammer Rheinland-Pfalz, Mainz, Germany, Ref. 2019–14162).

### Caliper-based navigation technique

**Fig 1:**
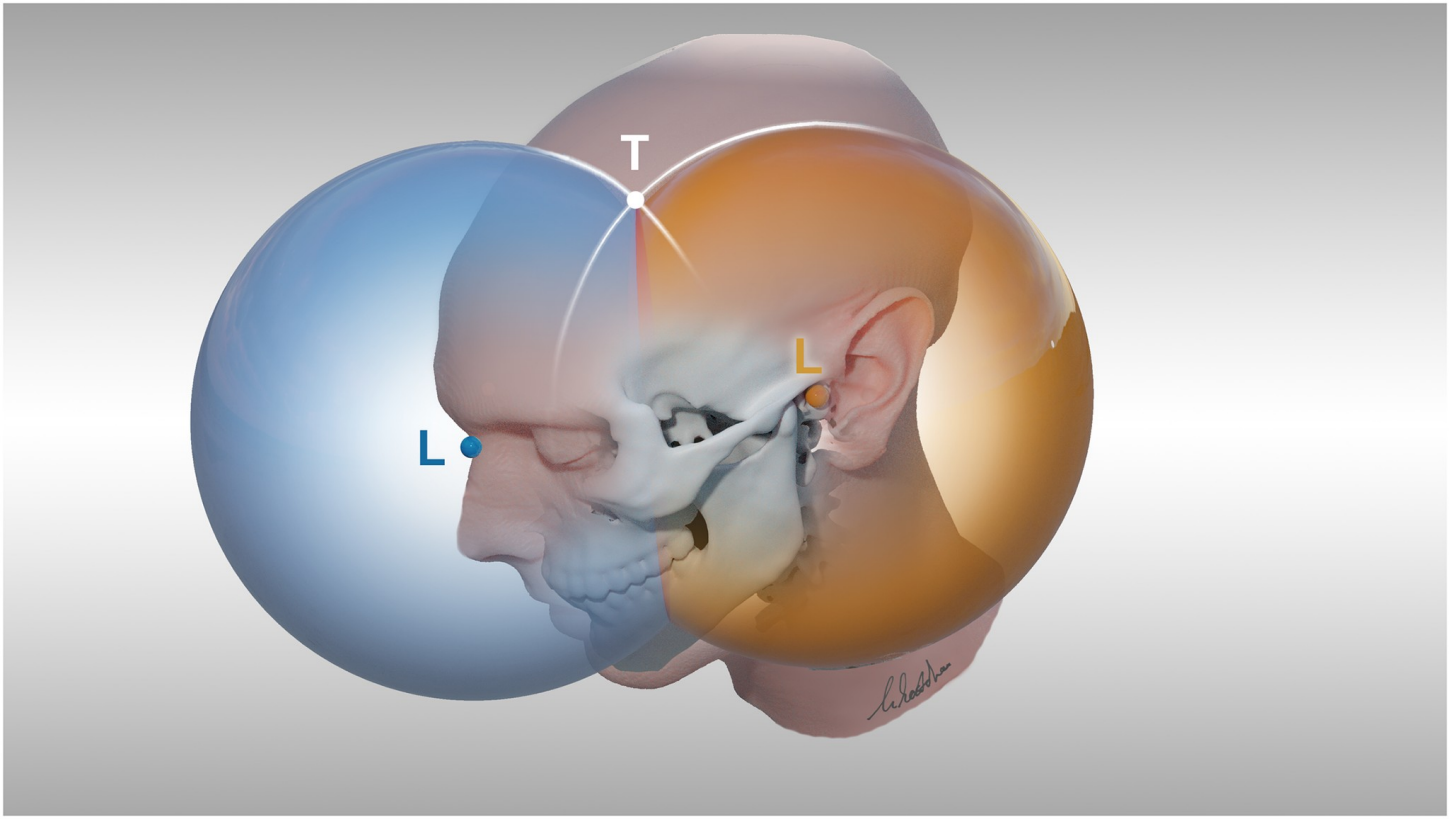
Scheme of the method. *Yellow L* Landmark Porus acusticus externus, *blue L* Landmark Nasion, *T* Target.

Briefly, for this technique a target is defined in an imaging dataset and the distances of the target from two anatomical surface landmarks are calculated. Then, circles with a radius equal to the distance from the landmark and centered around the landmarks are drawn onto the head of the patient with the help of a specially manufactured caliper. The intersection of both circles defines the target (Fig 1) The geometrical background is explained in full detail in the *Appendix* and a step-by-step guide is available as *video guide*.

The caliper navigation consists of two parts with the following prerequisites.

#### The calculating part

Prerequisites are conventional cuboid CT or MRI datasets, any image viewer, a conventional calculator.

#### The marking part

Prerequisite is a suitable caliper.

### Calculating part at the image viewer (Fig 2)

**Fig 2:**
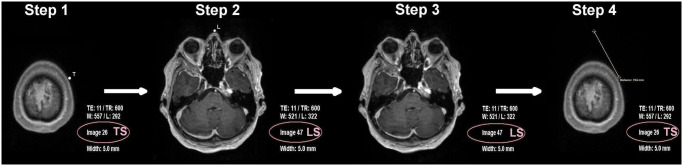
Calculating part. Assessment of the distance between the Landmark *L* and the target *T*, shown for nasion as landmark. For step-by-step explanations see paragraph *Calculating part* and the *video guide*. *TS* target slice, *LS* landmark slice.

**STEP 1** Define the target (T) in the image viewer software. T will be the center of the desired craniotomy correctly projected onto the skin surface for the respective lesion (see chapter *troubleshooting* for the correct choice of a target).
Memorize the running number of the respective target slice (TS).
**STEP 2** Scroll to the slice of the first landmark (L), nasion. Memorize the respective landmark slice (LS).
**STEP 3** The cursor function of the applied image viewer is set to *distance measurement*. Within the landmark slice (LS), the cursor is placed on L and will not be moved from this cursor position until step 4.
**STEP 4** Scroll back to TS without moving the cursor on the screen surface. The number of scrolled slices are multiplied by the slice thickness to assess the distance “travelled” by the cursor between LS and TS. This is distance *a*. Within TS, measure the distance from the priorly unchanged cursor position to T. This is distance *b*. The distance from L to T is $\sqrt{a^{2}+b^{2}}$ (Pythagoras’ theorem).

Perform the same steps for the second landmark, porus acusticus externus, referred to as *porus* in the following. The distances from nasion to the target and from porus to the target have thereby been assessed. Note that some image viewer offer automated distance measurement between points in different slices, rendering this part much faster.

### Marking part on the head of the patient (Fig 3)

**Fig 3:**
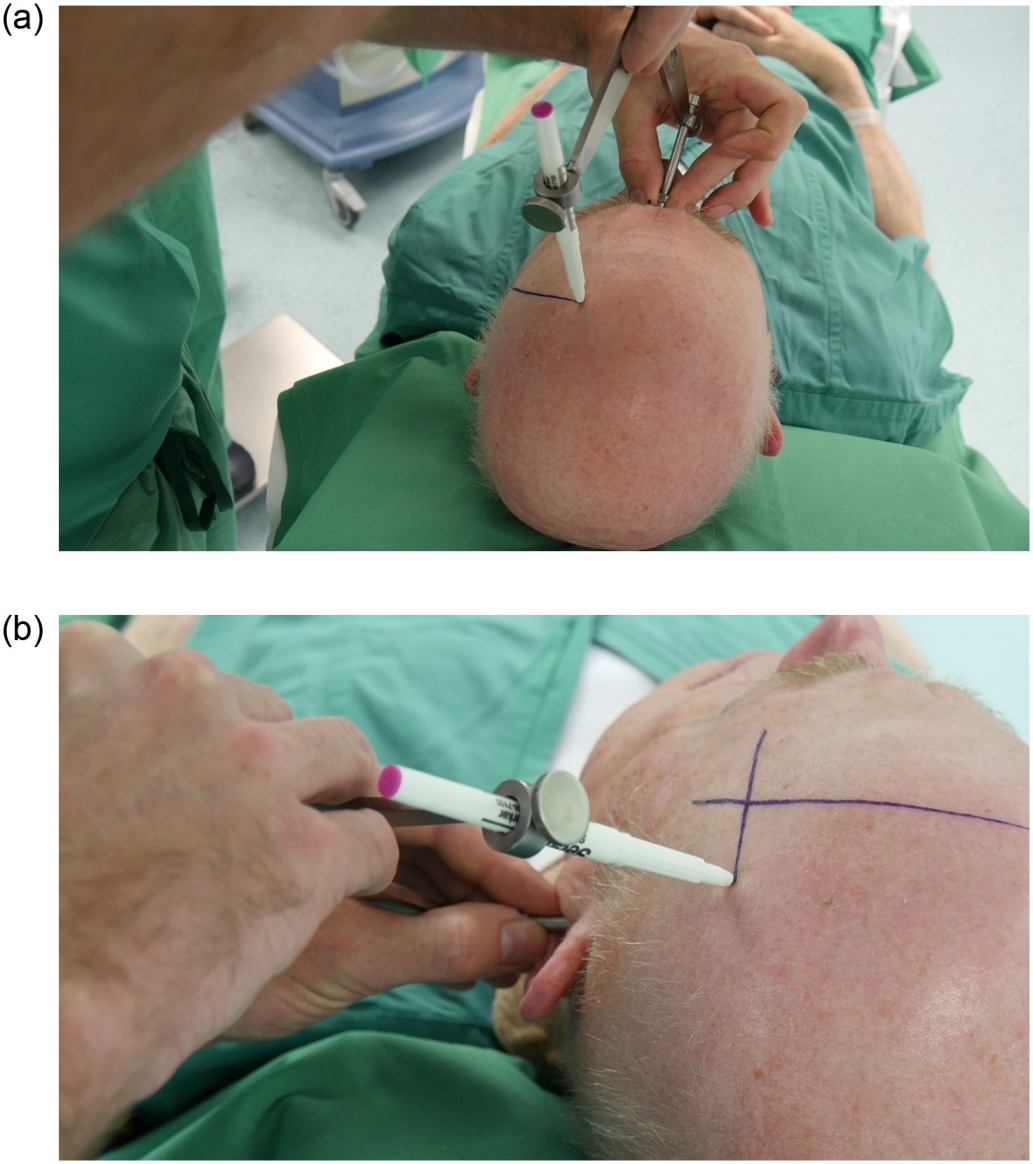
The distance from nasion to the target and the distance from porus to the target are marked onto the head with the help of a caliper. The individual in this manuscript has given written informed consent (as outlined in PLOS consent form) to publish these case details.

**STEP 5** The two respective distances are set on a caliper and circles are drawn for each landmark distance in the approximate target area on the patient’s head.
**STEP 6** The intersection of the two circles marks the target. If in doubt, a third landmark distance (e.g. bregma-target) can be calculated and used for control.

Open the video guide file (see S1 Video) with embedded audio narration that demonstrates the workflow of the caliper navigation technique. Both calculation part and marking part are illustrated in real time. The individual in this manuscript has given written informed consent (as outlined in PLOS consent form) to publish these case details.

### Validation of the caliper navigation

Validation of the caliper navigation technique was performed in two steps. In a first step, we sought for a model for preclinical evaluation and for obtaining data for a power analysis prior to the clinical evaluation. A saw bone skull was prepared with small screws placed over the convexity surface. The skull then underwent a CT scan and the caliper-based navigation technique was applied to localize the different targets defined by the screws. Additionally, the offset of computer navigation (Brainlab iplan, BrainLab Colibri Cart 1.1) to the real targets was assessed.

Afterwards, we conducted the second step, a clinical validation. Patients scheduled for cranial surgery assisted by computer navigation were included after informed written consent. Arbitrary targets on the convexity surface were chosen and marked in the computer navigation plan prior to surgery. In the operating theatre, each target was then first assessed by the caliper navigation. Afterwards, the target was located by a tracked pointer guided by the computer navigation, and the offset in mm between the two methods was registered-considering the navigated target position as the ground truth.

Statistical analysis and visualization were computed with Prism 9.0.2., Graph Pad Software, LLC., apart from the power analysis, which was carried out by the Department of Biometrics of our Institution. Data are expressed in mean ± standard deviation and 95% confidence intervals as 95% CI (lower limit; upper limit).

## Results

This study gives a step-by-step description of an alternative low-tech navigation technique, as well as its validation. The latter was practiced in two steps, first in a preclinical saw bone skull model and then in the clinical setting, with computer navigation as reference.

**Fig 4:**
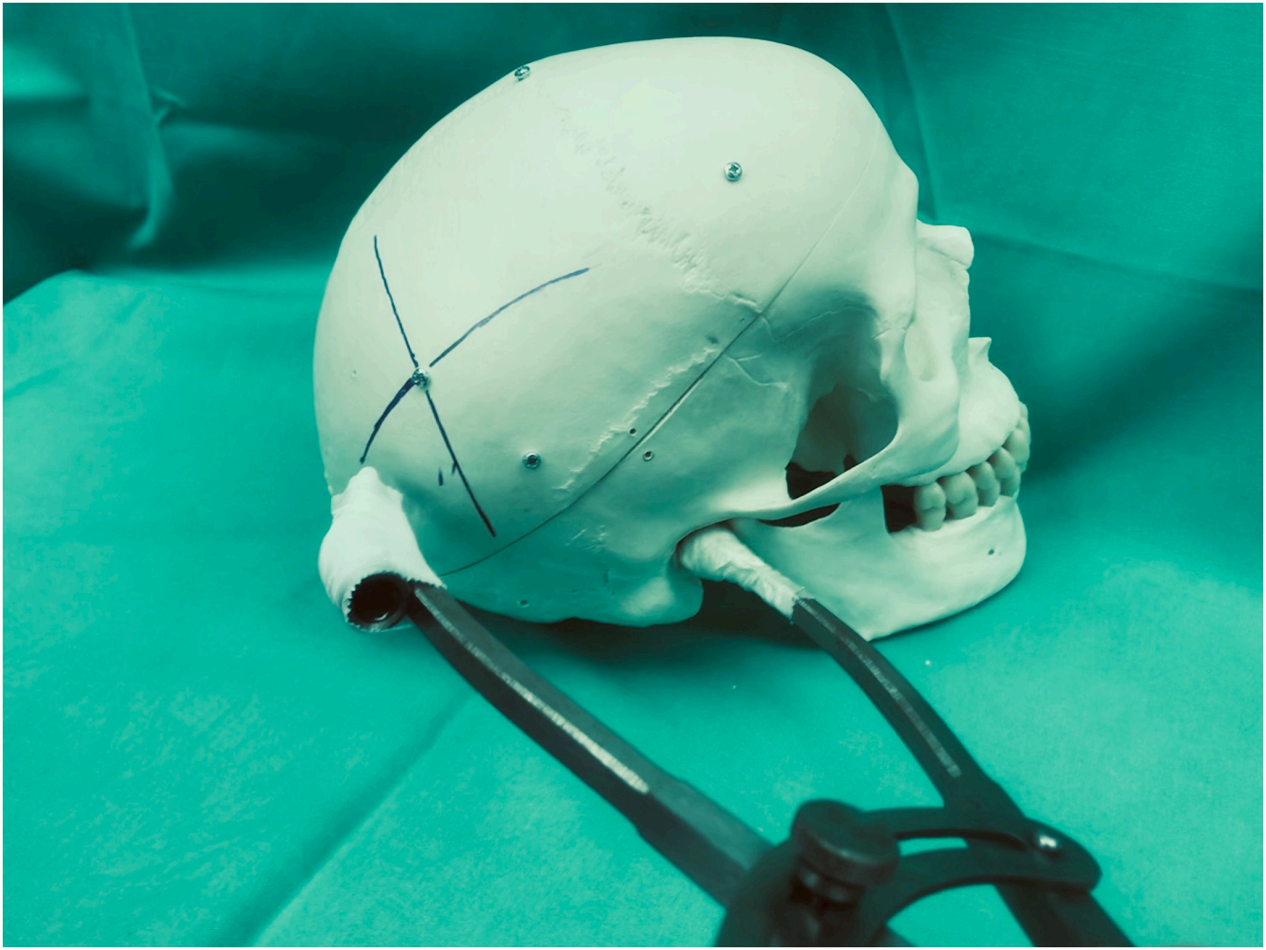
Example of the localization of one of the 20 targets on the saw bone model. The intersection of the two circles meets at the target.

**Fig 5:**
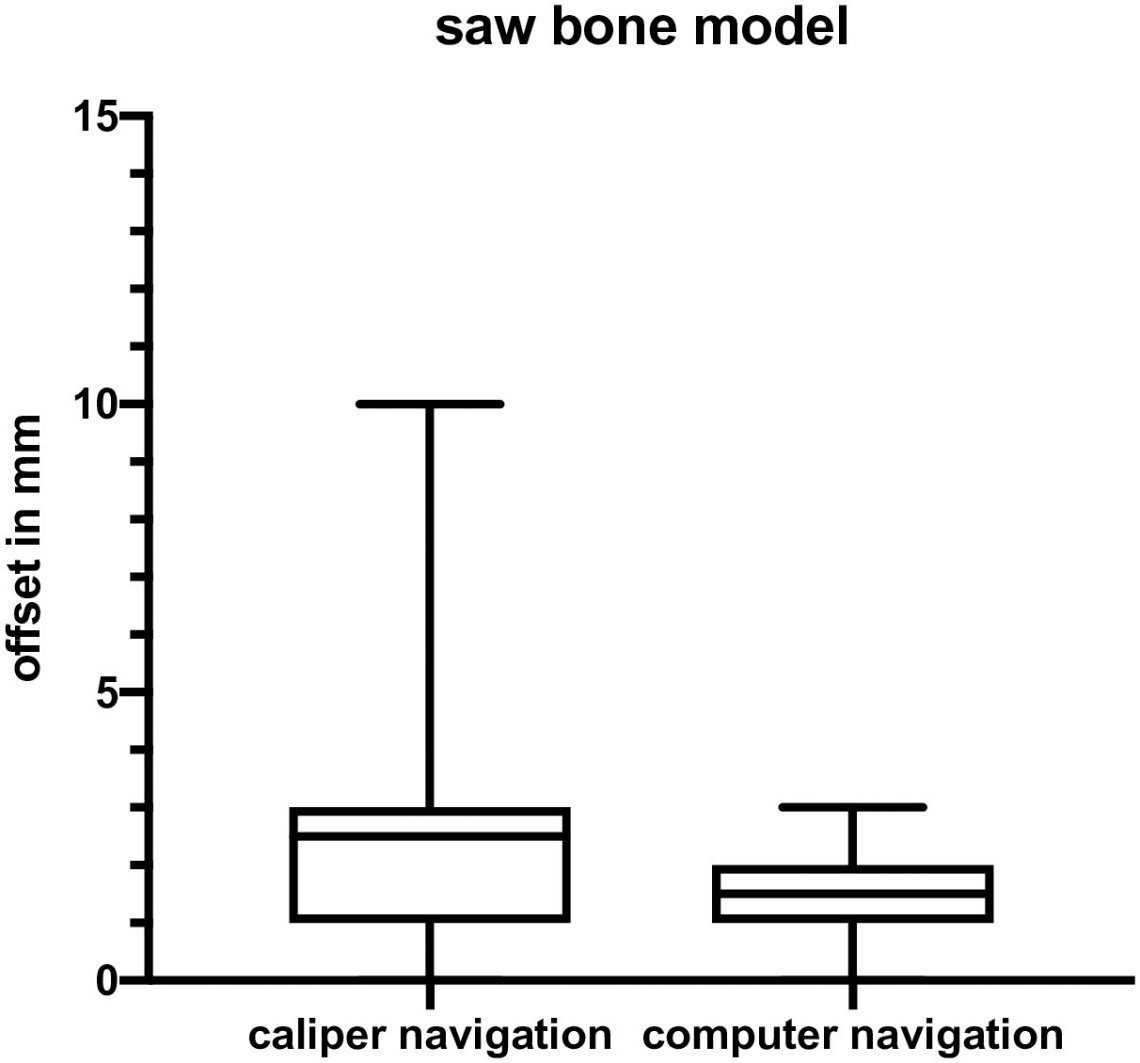
Box plot of data of the saw bone model, for caliper navigation and computer navigation. Whiskers: Max and min. Box: 25 and 75 percentiles and median.

In the saw bone skull model, 20 targets were assessed, localized at the frontal, temporal or parietal convexity. (Fig 4) The mean offset between the caliper-navigated target and the real target was 2.9 ± 2.8 mm in this series, 95% CI (1.6 mm; 4.2 mm). The same 20 targets were afterwards located by the computer navigation pointer. The mean offset between computer-navigated target and real target was 1.6 ± 0.9 mm in this model, 95% CI (1.2 mm; 2 mm). The difference between the two methods did not reach significance according to t-test (p = 0.06). (Fig 5).

Based on this data from the saw bone model, we computed a power analysis to find the required number of measurements to be performed in the clinical setting to validate the method (Bootstrap-95% confidence interval, expected 3-fold error in comparison to the saw bone model due to scalp imprecision, 1000 simulations). The required precision for validation was defined as an offset of not more than 13 mm. The derivation for this threshold will be explained in the discussion. This power analysis confirmed sufficiency of 100 targets to validate the method.

**Fig 6:**
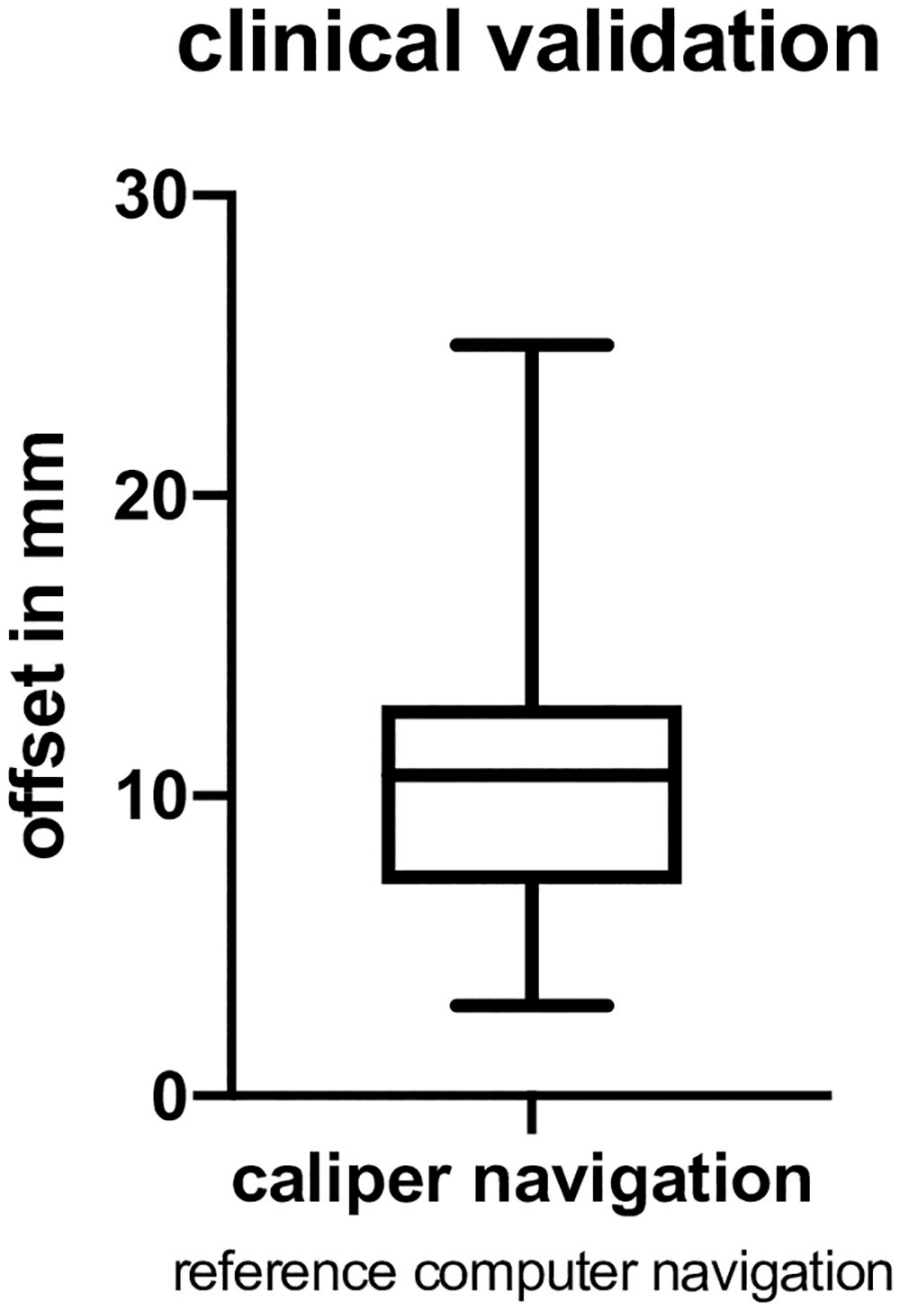
Box plot of data of the clinical validation, for caliper navigation, referenced to computer navigation as ground truth. Whiskers: Max and min. Box: 25 and 75 percentiles and median.

Hence, for clinical validation, 100 convexity targets in 15 patients were assessed with the caliper navigation, and their respective offsets were registered by computer navigation as ground truth, as described above. The mean offset to the target in reference to computer navigation was 11 ± 5.2 mm, 95% CI (9.9 mm; 12 mm).(Fig 6).

Neither the caliper navigation technique application itself nor running the study in the clinical setting caused any complications. The complete data of this work is available by following the PLOS ONE hyperlink.

## Discussion

This study assessed precision of a new and inexpensive navigation technique, by means of validation in a saw bone model as well as a clinical validation in the operating room. Our small sample of the controlled computer navigated target locations in the saw bone model is in line with manufacturer data. For real application on the head of the patient, precision is probably slightly reduced. Golfinos et al. report in their clinical analysis of 325 computer navigated cases a mean error ranging from 2.8 to 6.2 mm, depending on type of data set and on matching technique) [1].

The confidence interval of our data is within our set threshold of 13 mm (derivation of the threshold explained below). Yet, the offset variance was high with the method and 25% of the measured offsets were higher than allowed. We think that the highest outliers can be explained by maximal offsets in opposite directions of the two navigation methods.

Computer navigation is an indispensable tool for some and a very useful tool for many neurosurgical procedures, and with respect to its precision, we have used it as the reference for clinical validation of our caliper technique.

Nonetheless, due to its costs, computer navigation is a critical and limited resource. The setup remains somewhat time-consuming and navigation is therefore still not a routine tool in emergency surgery. Furthermore, a number of procedures do not require navigation during lesion resection (e.g. convexity meningioma or other superficial benign or metastatic tumors, limited epidural hematoma, burr holes for drainage of different hematomas) as long as the surgeon can rely to a reproducible and safe conventional method for craniotomy planning. The demand to the here presented caliper navigation was that it localizes targets and hence craniotomies precisely enough to carry out surgery.

Few teaching textbooks actually cover the technical aspect of how to locate a convexity target. Accordingly, it is the experience of the authors that even experienced neurosurgeons often do not have a methodologically precise technique for this task. Seeger et al. described several measurement and mapping techniques to choose from depending on the concerned convexity area. His approaches make use of the scan topogram [2]. The craniomapper and similar methods make use of a radiopaque mesh that is placed over the head during image acquisition [3]. These approaches require placement of radiopaque markers at the time of image acquisition, other techniques require additional radiographs of the head. Ikeda et al. have described a fairly complex model with several reconstructive planes and steps to perform [4]. Nakajema et al. published another algorithm of further complexity and hence would require integration of their software to the image viewer [5]. In our research of the literature and renown textbooks, we did not find a reported technique that does not require any preparation at the time of scanning or additional radiographs, any computed reconstruction of data and that has no demands to plane orientation.

The two-stepped validation shows a difference of precision of the caliper technique between the saw bone model with very high precision and the clinical setting with a higher offset. This is most likely due to the scalp mobility over the skull. The caliper fixation at the landmarks is never perfect, and hair can render the drawing more difficult. A great advantage of computer navigation is that precision can be controlled while surgically advancing from layer to layer, e.g. before skin incision and then again after retractor placement prior to the bony craniotomy. When performing craniotomies without computer navigation, special care must be taken to avoid an increasing misplacement from layer to layer (see *troubleshooting*).

In order to statistically confirm that precision with the caliper technique is sufficient, we had to define a precision threshold. Of course, such a threshold is somewhat arbitrary. We set this threshold to 13 mm based on the following assumption: In meningioma surgery, excision of dura is recommended 10 to 20 mm lateral to the tumor mass. Additional 5 mm are planned for dural patch suture. Surgeons will therefore shape the craniotomy approximately 25 mm larger to all sides than the meningioma mass. If the craniotomy is misplaced, then the procedure will still be feasible without difficulties if 10 mm of dura lateral to the mass can be excised and if 2 mm of dural margin are left for suture. Hence, a 13 mm offset (25 minus 12 mm) would be acceptable for convexity meningioma surgery. Other lesions that do not concern the dura allow higher offsets. Again, this method does not compete with computer navigation, but is presented as a fast, inexpensive and sufficient surrogate in suitable intracranial pathologies.

Application of Pythagoras’ Theorem to calculate a length between two points that lie in different radiological 2D slices is the base of computer image analysis software and has been described elsewhere [2,6]. Not all image viewers today supply this more sophisticated function to allow distance measurement between points in different slices, but every viewer routinely supplies a simple measurement function for distance measurement within the same slice-sufficient for the here presented technique.

**Fig 7:**
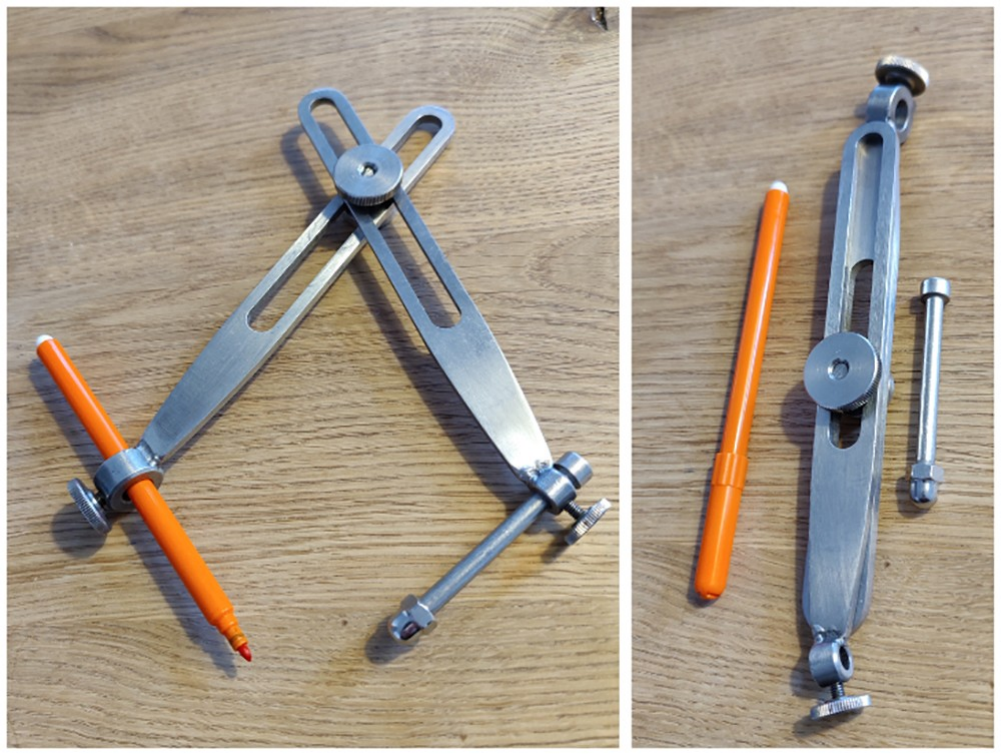
Craniotomy caliper. The demands to a caliper to facilitate the marking part of this method have been addressed in this model designed by the first author: The contact point of the center leg is smooth and can be inserted to the porus and placed on nasion. The length of legs of the caliper can be adjusted and together with the fix angle chosen for the mounting rings, this length adjustment allows to keep the angle of the center leg and the marker nearly vertical to the skin at all different convexity locations, which reduces the error. The mounting rings allow to insert different diameters of markers. The tool can be folded up and fits in a pocket.

The method works for any plane and with any landmark. We recommend the use of nasion and porus since they are easily identifiable and allow a relatively rigid fixation of the caliper. The use of a simple caliper elegantly overcomes the problem that the straight distance between landmark and target must be marked over the roundish head’s surface. In the course of this work, we developed a dedicated blunt caliper that corresponds to the shape and dimensions of a head, to felt-tip mounting and to error-limiting handling. (Fig 7) Nonetheless, the caliper navigation works with other types of dividers and calipers.

Initially, the calculating part may appear cumbersome, but it refers to easy fundamental geometry. The learning curve is steep and the technique time saving after few trials (see *online video*).

### Limitations

The clinical validation was carried out with reference to computer navigation as ground truth. The precision of the gold standard itself could not be quantified. We paid attention to best possible surface matching results of the computer navigation, but as is known, even computer navigation does not always offer the same precision all over the head. The true precision of the presented caliper navigation might therefore be slightly worse or better.

The slice thickness for the computer navigation used here was 1 mm. This is a data requirement for the use of navigation. For the caliper-based technique, we chose axial series with 3–5 mm slice thickness, because these conventional datasets will always be available. Furthermore, it facilitates counting the slices. Again, the aim was to validate whether the technique succeeds under basic conditions.

It is mandatory that the data set is cuboid to use this method. This is usually the case in MRI, but might have to be reconstructed at the CT console for some newer CT that apply tilt to the gantry.

## Conclusion

We have presented and validated an easy and inexpensive method that is sufficiently precise to plan a convexity craniotomy over a superficial lesion.

## Appendix

Geometrical Explanation of the caliper navigation technique.

### Calculating part

**Fig 8:**
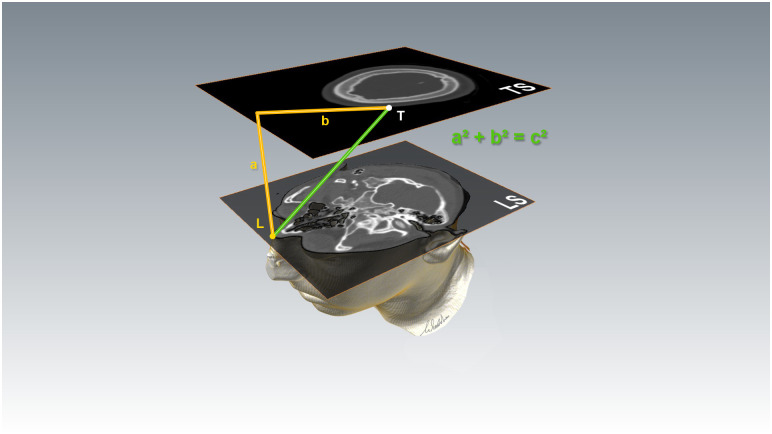
As long as the cursor position on the screen is not changed, the line *a* “travelled” by the cursor from landmark slice LS to target slice TS is vertical to the slice plane orientation. Hence, it is also vertical to the line *b*. The distance between L and T can therefore be calculated using Pythagoras’ theorem.

When the screen position of the cursor is not changed while scrolling through the slices, then the line *a* “travelled” by the cursor from TS to LS is vertical to all slice planes. Since the line *b* is within the slice orientation, *a* is vertical to *b*. Hence, the distance between L and T is $\sqrt{a^{2}+b^{2}}$, according to Pythagoras’ theorem. (Fig 8) It is of no importance whether the data set is exact in terms of axial, sagittal or coronal orientation or oblique (e.g. rotated head in obscured patients in emergency scans).

Any point identifiable on both imaging and the surface of the patient’s head can be used as landmark (e.g. bregma, inion, tuber parietale, occipital protuberance, palpable bone lesions). The use of nasion and porus has the advantages of easy localization, solid placement of the caliper with minimization of scalp shift over the skull while marking, and the fact that for most convexity areas, the two circles will intersect in an obtuse angle. This all offers the best accuracy for the presented technique.

**Fig 9:**
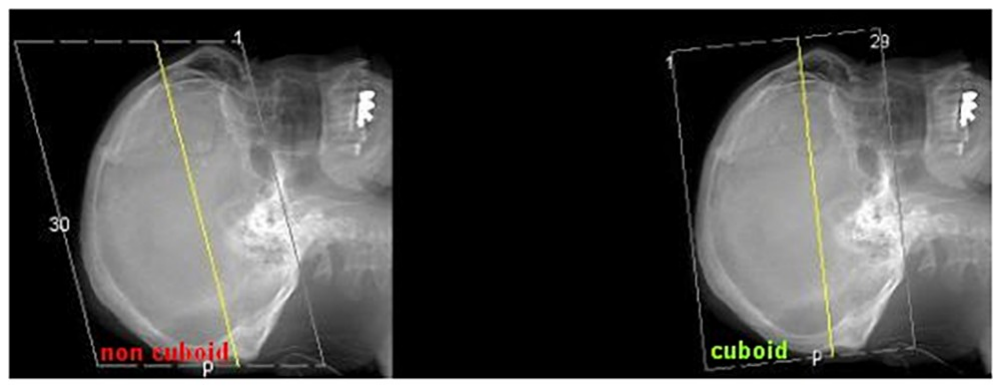
In order to be able to apply Pythagoras’ theorem, the line “travelled” by the cursor through the slices must be vertical to the slice orientation. This is only the case if the slices are “piled aligned” in a cuboid dataset (*right*). If the gantry angle is modified during data acquisition, the resulting dataset is a non-cuboid parallelepiped, and the calculation cannot be applied. In that case, a cuboid dataset can be reconstructed at the CT workstation. Whether the regarded slices are applicable for the caliper navigation technique or not can be seen at a glance in the corresponding scout.

However, it is mandatory that the dataset used for the calculation is cuboid. Some CT scanner can tilt the gantry, which is employed to modify the field of acquisition to reduce irradiation to the eyes during head CTs. If such an acquisition technique is used, then an additional cuboid dataset must be reconstructed at the CT. This can be done without new acquisition and irradiation. The gantry angle can be seen in the scout of the corresponding dataset (*see* Fig 9).

### Marking part

The calculation part has delivered the lengths of the straight lines between two identifiable landmarks and the target. Geometrically, in a three-dimensional space, the position of a point (in our case the landmark L) and a length (in our case the distance from L to T) defines a ball. The target T can be anywhere on the ball’s surface.

Furthermore, it is known that the target has been chosen on the surface of the head. Because the head is a spherical structure, the easiest way is to use a caliper set to the respective length. The caliper drawing will immediately indicate the common points of this geometrical ball with the head surface, a ring-like structure on the surface of the patient. The target T can hence be anywhere on this drawn structure. When adding a second ball centered around a second landmark L, the second intersective structure between the surface of that second respective ball and the surface of the head again can be drawn. The intersection of these two structures is the target. (see Fig 1).

It should be mentioned that geometrically in most cases, the two drawn ring-like structures intersect in a second point on the patient’s surface. However, the second intersection is usually far off the target and can be ruled out by common sense, since it would project to the chin or neck area in most cases. If the correct one cannot be identified by common sense, a third distance from a separate landmark (e.g. bregma) can help.

### Troubleshooting

#### Definition of the target

The correct target at the skin surface for a certain intracranial lesion is chosen by planning from inside to outside. A straight line from the center of the most superficial part of the lesion which projects vertically to the nearest dural and bony layers and through the scalp, indicates the correct target.

**Fig 10:**
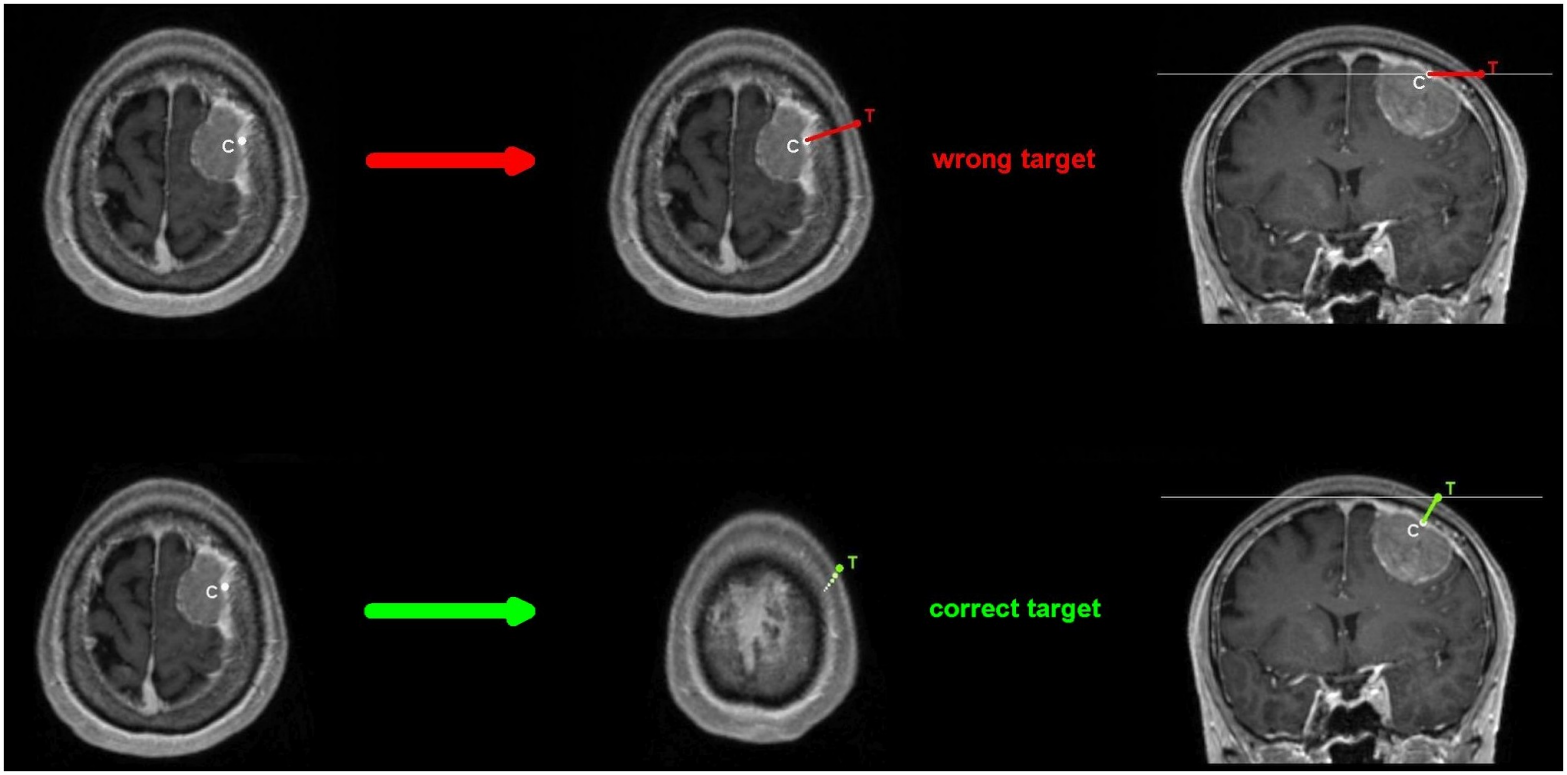
*Top*. A common error occurs when the target on the skin surface is chosen from a single plan, here axial orientation. This approach leads to a wrong center of the craniotomy for the respective lesion. *Bottom*. A corrective approach is to move and scroll with respect to the slice thickness to obtain an oblique trajectory.

A common error occurs when the target is chosen from a single plane view by choosing a wrong target slice by “sticking to the lesion”. The lesion is not necessarily visible in the correct target slice (Fig 10. *top*, *wrong target*). The target should be defined from two planes, eg. axial and coronal, whenever possible.

If only one plane orientation can be used, a fairly quick way to keep the target centered for the respective lesion is:

1. Place the cursor at the central point of the lesion at dural level.
2. Scroll back and forth between all slices that contain the lesion without moving the cursor. If the cursor remains approximately at dural level, the line to the skin surface to mark the target can be drawn in the same slice. If the cursor changes to deeper and more superficial layers while scrolling back and forth through the slices containing the lesion, then the line to the closest surface must be established stepwise—scroll and move-with reference to the respective slice thickness-until the cursor hits the skin surface to set the target at that position. (Fig 10. *bottom*, *correct target*)

**Fig 11:**
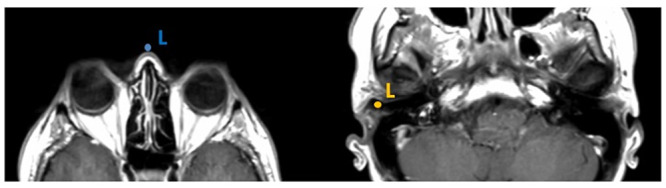
*Left*, *blue Landmark L*, *nasion*: The slice that contains the center of both eyeballs, shown by presence of the lenses. *Right*, *yellow landmark L*, *porus*: The slice containing the outer part of the external auditory canal is chosen and the point is placed medial to the tragus. This is the position where the blunt leg of the caliper will come to rest.

Ensure the correct and reproducible identification of the landmarks and the target in the images. We recommend nasion and porus as landmarks for the reasons mentioned above. (Fig 11).

Do not move the cursor while scrolling through the slices between LS and TS.

Be familiar with the calculator used. Function differs from product to product. Calculation errors will typically lead to large offsets and can be immediately ruled out by common sense.

The scalp moves over the bone. Ensure that draping does not cause displacement of the marked scalp. Once draped, no further remeasurements are possible. Whenever possible, chose a straight incision twice as long as the desired craniotomy, centered through the target. Symmetrical mobilization of the scalp to both sides followed by retractor placement with moderate opening pressure will keep the target in the center of the exposed bone and will limit the loss of precision of the following craniotomy and durotomy work.

## Supporting information

S1 File Minimal data clinical validation. (PDF)

S2 File Minimal data saw bone. (PDF)

S1 Video Video-guide-caliper-navigation-blinded-20MB.mp4. (MP4)
