# Supplementary material for: Caliper navigation for craniotomy planning of convexity targets
Source: PLoS One. 2021 May 20;16(5):e0251023. doi: 10.1371/journal.pone.0251023 (PMC8136664; doi:10.1371/journal.pone.0251023)
Supplement: S1 File — (PDF) [file pone.0251023.s001.pdf]

clinical validation caliper navigation

Number of values		100
Minimum		3
25% Percentile		7.05
Median		10.7
75% Percentile		13
Maximum		25
Range		22
Mean		10.96
Std. Deviation		5.221
Std. Error of Mean		0.5221
Lower 95% CI of mean		9.92
Upper 95% CI of mean		11.99
Shapiro-Wilk test		
W		0.9369
P value		0.0001
Passed normality test (alpha=0.05)?	No	
P value summary	***	
Test for lognormal distribution		
Shapiro-Wilk test		
W		0.9816
P value		0.1781
Passed lognormality test (alpha=0.05)?	Yes	
P value summary	ns	
Number of values		100
Impossible values in lognormal distributions		
Number of zeroes		0
Number of negative values		0

**minimal data**	**Patient Nr**		**running target nr**	**offset in mm**
all offsets of caliper navigation		1	1	6.8
referenced to computer navigation			2	6.8
as ground truth			3	12.1
			4	4.3
			5	10.7
			6	7.6
		2	7	9.4
			8	11.6
			9	5.7
			10	13.6
		3	11	8.2
			12	5.6
			13	7.4
			14	3.4

4	15	6.2
	16	4.7
	17	17.6
	18	11.3
5	19	12.9
	20	14.2
	21	14.2
	22	16.3
	23	8
	24	9
6	25	5
	26	8
	27	7.6
	28	3.5
	29	11.5
	30	11.4
	31	5.6
7	32	11.3
	33	5.6
	34	5.5
	35	7.5
	36	10.9
8	37	12.2
	38	7.5
	39	10
	40	7.2
	41	7
	42	10.7
	43	4.8
	44	7.2
9	45	7
	46	20
	47	12
	48	9
	49	12
	50	9
10	51	3
	52	6
	53	12
	54	8
	55	6
	56	3
	57	10
	58	15
	59	8
	60	8
11	61	12
	62	8
	63	11
	64	4
	65	12
	66	11

	67	7
	68	11
	69	5
	70	12
12	71	6
	72	10
	73	15
	74	15
	75	20
	76	23
	77	17
13	78	21
	79	13
	80	4
	81	23
	82	12
	83	9
	84	13
	85	14
14	86	12
	87	11
	88	10
	89	19
	90	17
	91	25
	92	21
	93	22
	94	24
	95	23
15	96	17
	97	16
	98	13
	99	12
	100	10
