# Supplementary material for: Caliper navigation for craniotomy planning of convexity targets
Source: PLoS One. 2021 May 20;16(5):e0251023. doi: 10.1371/journal.pone.0251023 (PMC8136664; doi:10.1371/journal.pone.0251023)
Supplement: S2 File — (PDF) [file pone.0251023.s002.pdf]

saw bone model

	caliper navigation	computer navigation
Number of values	20	20
Minimum	0	0
25% Percentile	1	1
Median	2.5	1.5
75% Percentile	3	2
Maximum	10	3
Range	10	3
Mean	2.9	1.6
Std. Deviation	2.808	0.9403
Std. Error of Mean	0.6279	0.2103
Lower 95% CI of mean	1.586	1.16
Upper 95% CI of mean	4.214	2.04
Paired t test		
P value	0.0569	
P value summary	ns	
Significantly different (P < 0.05)?	No	
One- or two-tailed P value?	Two-tailed	
t, df	t=2.028, df=19	
Number of pairs	20	

	caliper navigation	computer navigation
minimal data (offset in mm)	10	2
	3	2
	1	1
	0	0
	2	3
	1	2
	3	1
	5	1
	1	3
	3	1
	3	1
	1	3
	5	1
	10	2
	3	1
	1	1
	0	0
	2	3
	1	2
	3	2
